# The Forgotten Healer: The Role of Adipose Tissue in Spontaneous Healing After Free Flap Finger Reconstruction

**DOI:** 10.3390/jpm16020110

**Published:** 2026-02-12

**Authors:** Macarena Vizcay, Giorgio E. Pajardi, Alessandro Mastroiacovo, Luigi Troisi

**Affiliations:** 1 IRCCS Multimedica Group, University Department of Hand Surgery & Rehabilitation, San Giuseppe Hospital, 20123 Milan, Italy; giorgio.pajardi@unimi.it (G.E.P.); luigi.troisi@unimi.it (L.T.); 2 School of Specialization in Plastic, Reconstructive and Aesthetic Surgery, Milan University, 20122 Milan, Italy

**Keywords:** adipose tissue, fingertip injury, great toe pulp flap, trimmed great toe flap, hand reconstruction, fingertip reconstruction, fat viability, wound healing

## Abstract

**Background:** Digital pulp reconstruction with toe-based flaps reliably restores sensibility and contour, yet the healing behavior of viable subcutaneous fat remains underexplored. Because adipose tissue exhibits patient-specific regenerative and volumetric responses, its preservation represents a key element of personalized fingertip reconstruction. This study evaluates the outcomes of toe pulp flaps with targeted fat preservation to assess how individual tissue biology influences contour and functional recovery. **Methods:** We retrospectively reviewed consecutive digital reconstructions performed with free toe flaps and several variations (pulp toe flap, chimeric pulp toe flap, trimmed great toe flap and chimeric pulp+ trimmed great toe). Particular attention was given to healthy subcutaneous fat that was deliberately maintained or exposed to help shape the final contour. All patients were followed clinically and photographically until complete healing occurred. **Results:** A total of 126 patients underwent a finger reconstruction with free toe flaps and several variations. The preserved fat layer was intentionally left exposed to promote healthy granulation and spontaneous epithelialization, contributing favorably to the final contour of the distal pulp as the nail advanced. All wounds healed without the need for skin grafts. All patients achieved good to excellent functional and esthetic outcomes with minimal donor-site morbidity. **Conclusions:** This large retrospective series confirms the reliability of a healthy flap to help shape the digital reconstruction, highlighting the regenerative potential of viable digital fat. Incorporating this concept into the flap design may reduce the need for grafting, minimize donor-site morbidity, and enhance reconstructive outcomes in hand surgery.

## 1. Introduction

Digital amputations have increased in recent years, not only in low- to middle-income countries (LMICs) but also in high-income settings [1,2]. This rise reflects ongoing occupational hazards, road traffic accidents, domestic injuries, and recreational trauma. Beyond the immediate physical loss, digital amputation carries a profound psychosocial burden. Given the visibility of the hand in daily interpersonal interactions, even partial digital loss may lead to social withdrawal, altered self-image, and reduced quality of life [3]. These effects are particularly pronounced in young and working-age individuals for whom hand appearance and function are closely tied to personal identity and professional activity.

From a functional perspective, the hand represents one of the most complex and highly specialized units of the human body. Fine motor control, strength, precision grip, and tactile sensibility depend on the integrated interaction of bone, tendon, nerve, skin, and subcutaneous tissues. Even minimal tissue loss at the fingertip may result in disproportionate impairment, affecting dexterity, endurance, and object manipulation [4]. For this reason, reconstructive planning following digital amputation must be approached with particular care, requiring a patient-specific balance between durability, sensibility, contour, and esthetic integration. In this context, digital reconstruction should not follow a standardized model but rather a personalized, “tailor-made” strategy that reflects the individual anatomical, functional, and biological characteristics of each patient [5].

For these reasons, reconstruction should not be guided solely by functional considerations, but should also address esthetic restoration, which plays a critical role in patient satisfaction, social reintegration, and long-term acceptance of the reconstructed digit [5]. The volar pulp contributes more than half of the fingertip volume and plays a fundamental role in grip stability, proprioception, tactile discrimination, and shock absorption. Loss of this specialized soft tissue compromises not only mechanical performance but also sensory feedback, underscoring the importance of restoring adequate pulp bulk and compliance during reconstruction [6].

Over recent decades, a wide range of reconstructive techniques has been described for fingertip and complete digital defects, including local flaps, regional flaps, distant flaps, composite grafts, and microsurgical tissue transfer [7,8,9,10,11]. However, no single approach is universally applicable, and the choice of technique must be tailored to defect size, location, mechanism of injury, and patient-specific factors. In this context, the principle of “like-with-like” reconstruction remains central. Owing to its anatomical, histological, and biomechanical similarities to the hand, the foot—particularly the toe pulp—represents an excellent donor site for digital reconstruction [12,13]. Toe-based free flaps reliably restore sensibility, durability, and contour, and have become a cornerstone in modern fingertip and digital reconstruction.

While considerable attention has been devoted to flap survival, sensibility, and nail complex reconstruction, comparatively little focus has been placed on the biological behavior of subcutaneous adipose tissue within these flaps. Traditionally, adipose tissue has been regarded as a passive filler, frequently trimmed, buried, or discarded to facilitate skin closure. In contrast, emerging evidence from regenerative medicine and reconstructive surgery has demonstrated that adipose tissue is a metabolically active organ that is rich in adipose-derived stromal cells, vascular networks, and paracrine mediators that are capable of influencing angiogenesis, inflammation, and tissue remodeling [14,15,16].

In this study, we retrospectively analyze a large consecutive series of free toe-based digital reconstructions, with specific attention paid to the healing behavior of preserved subcutaneous fat. We aim to explore the role of adipose tissue as a biological adjunct in fingertip reconstruction, hypothesizing that viable digital fat acts as an active scaffold supporting spontaneous healing, contour optimization, and stable reconstruction while facilitating early rehabilitation and minimizing donor-site morbidity.

## 2. Materials and Methods

We conducted a retrospective review of consecutive digital reconstructions performed using free toe-based flaps from 2019 to 2025. The inclusion criteria were patients older than 18 years old undergoing digital reconstruction with free toe tissue for traumatic or post-traumatic defects of the fingers. Patients with incomplete clinical records or insufficient postoperative follow-up were excluded.

Reconstruction was performed using several free toe flap variations, selected according to defect characteristics and reconstructive requirements. These included the pulp toe flap, chimeric pulp toe flap, trimmed great toe flap, and chimeric pulp plus trimmed great toe flap. Flap design and composition were tailored to defect size, location, and functional demands, with particular emphasis placed on restoring digital contour and sensibility. Detailed descriptions of the surgical techniques have been previously published and are therefore not reiterated in the present study [12,13,17].

A specific focus of this study was the management of subcutaneous adipose tissue. In all cases, healthy digital fat was intentionally preserved, incorporated and exposed with the aim of enhancing the soft-tissue contour, supporting spontaneous healing, and minimizing the donor site morbidity. Fat handling strategies were documented intraoperatively, including the extent of fat preservation and its relationship to final wound closure.

Patients’ medical records were reviewed and included demographics, smoking status, presence of risk factors, type of trauma, flap reconstruction, dimensions, reoperations, operative time, ischemia time and long-term complications. Particular attention was given to the healing behavior of areas where subcutaneous fat was deliberately maintained or exposed. All patients were followed both clinically and photographically at regular intervals until complete wound healing was achieved. Serial photographic documentation was used to assess progressive epithelialization, contour evolution, and the overall esthetic outcome.

The primary outcome was complete healing, defined as full epithelial coverage of the recipient site without the need for additional surgical intervention. Spontaneous epithelialization over adipose tissue was defined as progressive epithelial coverage of intentionally exposed viable adipose tissue, achieved without skin grafting or secondary flap procedures. Healing was assessed clinically during follow-up visits. Time to healing was defined as the interval between surgery and documentation of complete epithelialization.

Donor-site healing was considered to be uneventful when it occurred without complications, defined by the absence of infection, wound breakdown, chronic pain, or functional impairment, including gait disturbance or limitations in daily activities.

Patients were evaluated at regular postoperative intervals (typically every 7 days after post-operatory discharge until complete healing of the finger and the donor site. Then, follow-up followed a 1–3–6–12 months visit pattern and all patients had at least 1 year of follow-up.

Complications were recorded based on clinical documentation and included: partial flap necrosis, total flap loss (defined as complete flap failure requiring revision surgery), infection, (defined as clinical signs of infection requiring antibiotic treatment), wound dehiscence, (defined as separation of the wound edges after initial closure), and donor-site complications, (including delayed healing, nail deformity, or functional complaints).

The statistical analysis was descriptive. Continuous variables are reported as mean ± standard deviation or median with range, as appropriate. Categorical variables are presented as counts and percentages. Missing data were handled by case-wise exclusion for the specific variable analyzed. No inferential statistical testing was performed.

## 3. Results

A total of 126 patients underwent fingertip reconstruction using toe-based free flaps and their variations, including 113 males (89.7%) and 13 females (10.3%). Reconstruction was most commonly performed using great toe pulp (GTP) flaps (71.4%), followed by trimmed great toe (TGT) flaps (14.3%), chimeric toe flaps (7.1%), and second toe-based reconstructions (4.0%). Tobacco use was reported in 15.9% of patients.

The mean operative duration was 266.8 ± 82.3 min (range, 98–470), and the mean length of hospital stay was 5 days. Complications included partial flap necrosis in 9 patients (7.1%) and long-term nail deformities in 2 patients (1.6%). Complete healing of the reconstructed digit was achieved within a mean of 4–5 weeks.

In all free flap reconstructions, adipose tissue harvested from the donor site was systematically preserved and used to tailor and contour the flap. To illustrate the versatility and technical nuances of flap utilization, three representative cases are presented, highlighting the different reconstructive scenarios and applications of the technique.

### 3.1. Case 1—Use the Fat as a Nailbed

**Figure 1:**
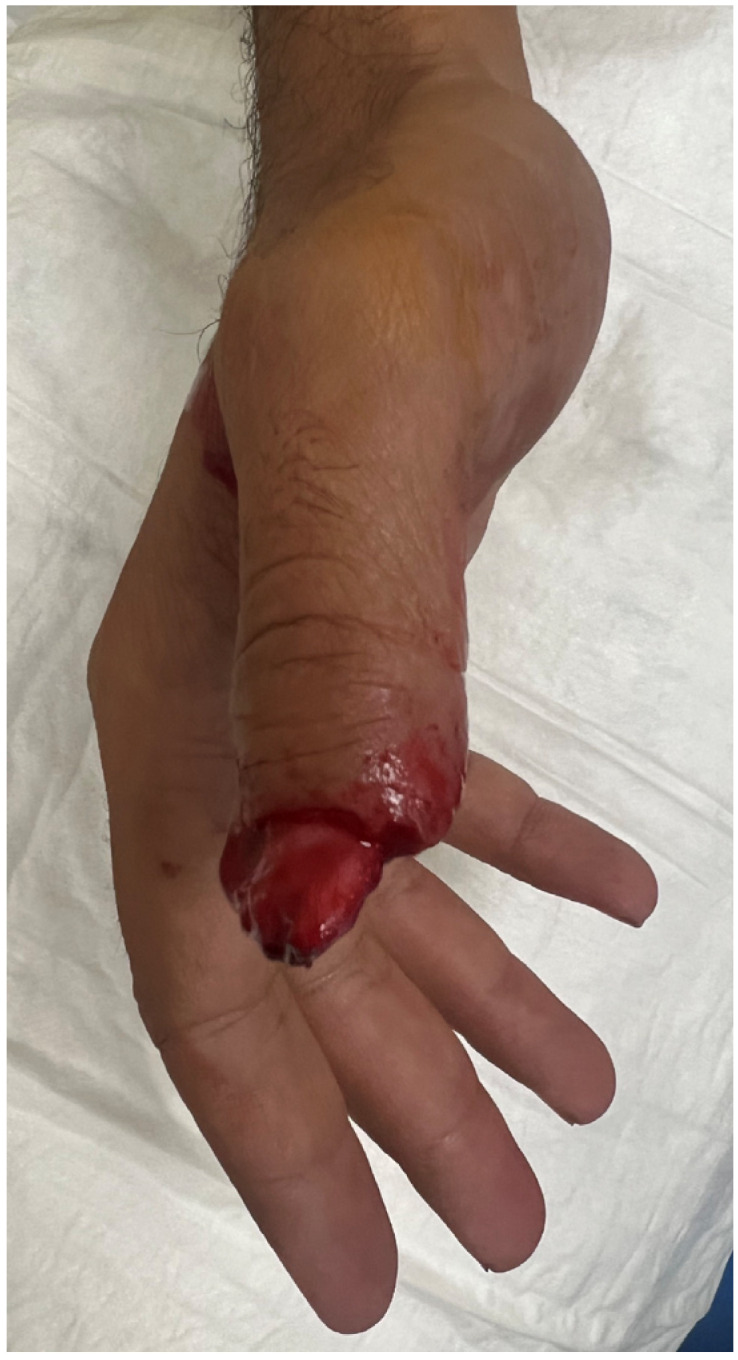
Traumatic amputation of tip thumb on the dorsal view.

**Figure 2:**
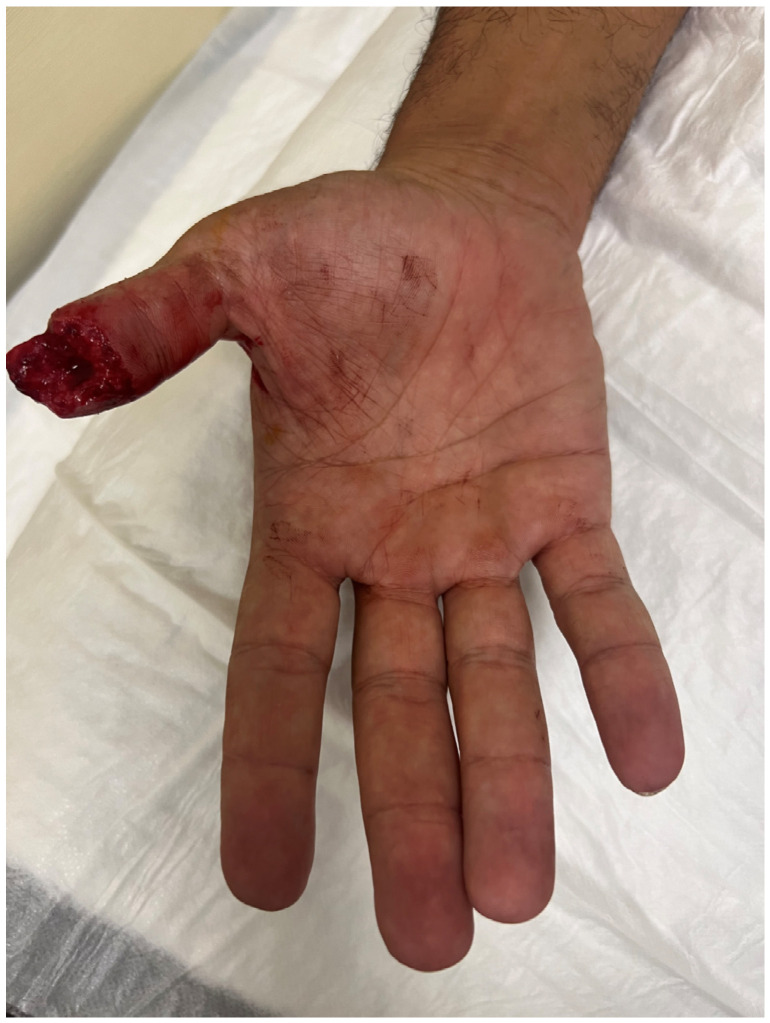
Traumatic amputation of tip thumb on the volar view.

**Figure 3:**
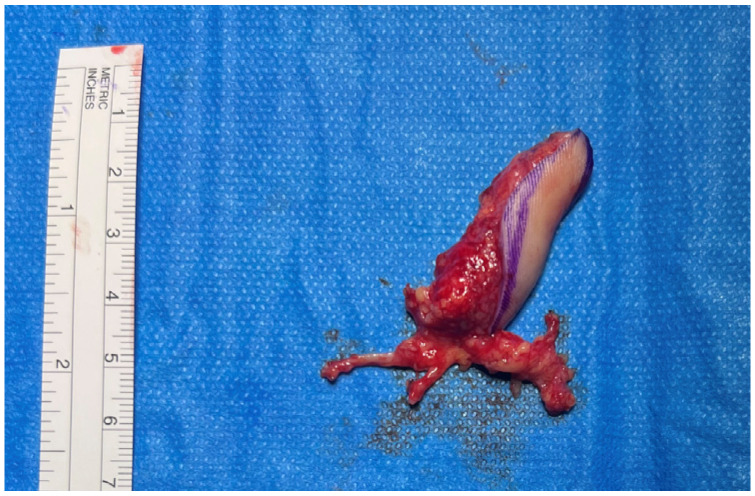
Great toe pulp free flap with fat pad.

A 40-year-old male presented with a traumatic distal thumb tip amputation, characterized by exposed bone and loss of the nail complex. Given the extent of soft-tissue loss and the functional importance of the thumb, reconstruction was planned using a free great toe pulp flap to restore both the volar padding and dorsal contour while preserving sensibility (Figure 1, Figure 2 and Figure 3).

**Figure 4:**
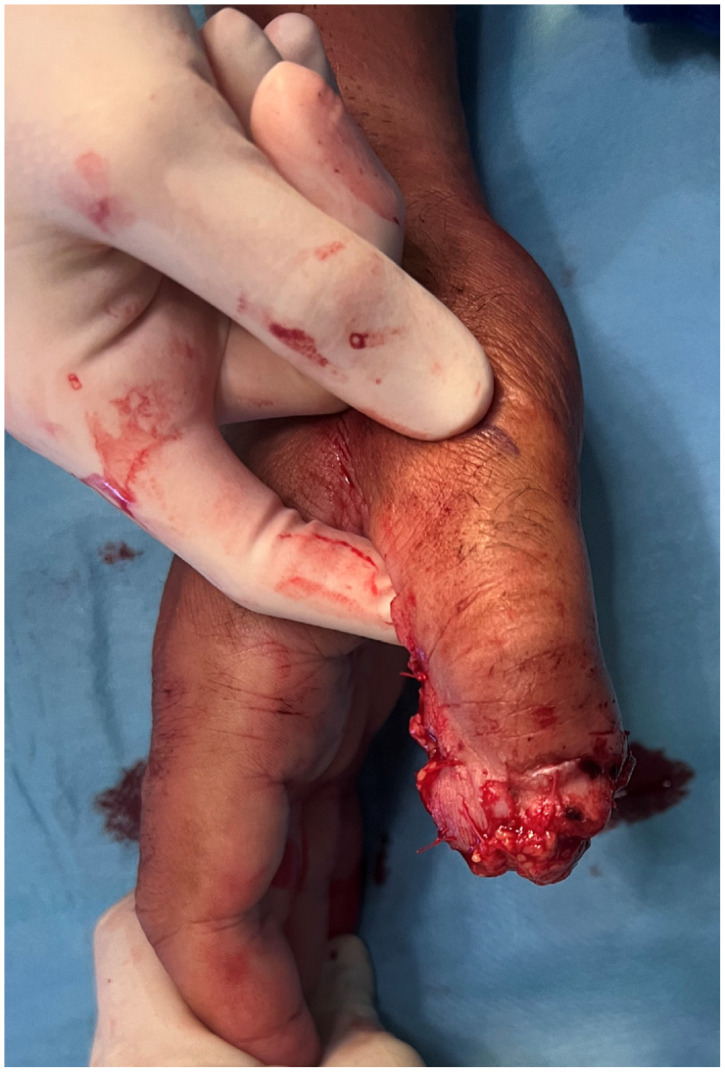
The flap inset on the dorsal view, using the fat as the nail bed.

**Figure 5:**
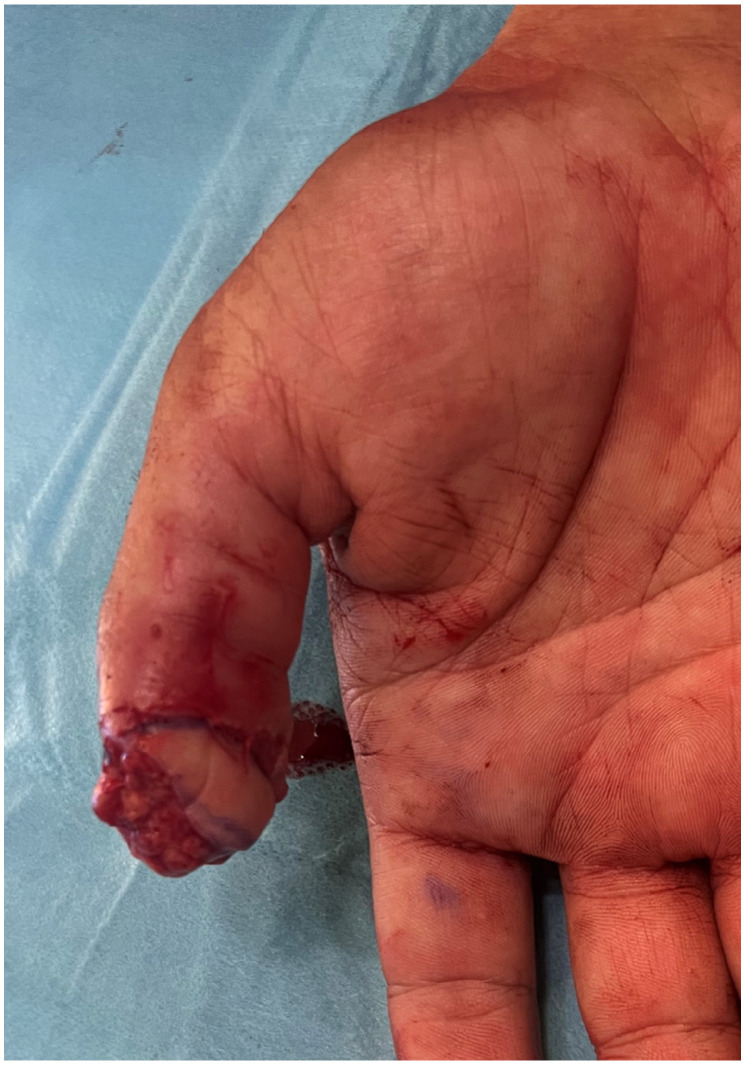
The flap inset on the volar view, using the skin pad on the pulp.

The flap was harvested from the fibular aspect of the great toe and included a well-vascularized adipose component in addition to the pulp skin (Figure 3). Particular care was taken to preserve the integrity of the subcutaneous fat during harvest, recognizing its potential role in contour restoration and secondary healing. During flap inset, the adipose tissue was intentionally positioned dorsally to recreate a compliant soft-tissue bed, serving not only to restore volume but also to function as a surrogate nailbed. The pulp component of the flap was oriented volarly to reconstruct the thumb pad (Figure 4 and Figure 5).

Postoperatively, the flap demonstrated uneventful healing with progressive integration of the adipose tissue into the dorsal aspect of the thumb. The patient was allowed to resume ambulation with the use of protective footwear for the first 3 weeks and then initiated hand and foot washing after, without compromising the flap viability or donor-site healing.

**Figure 6:**
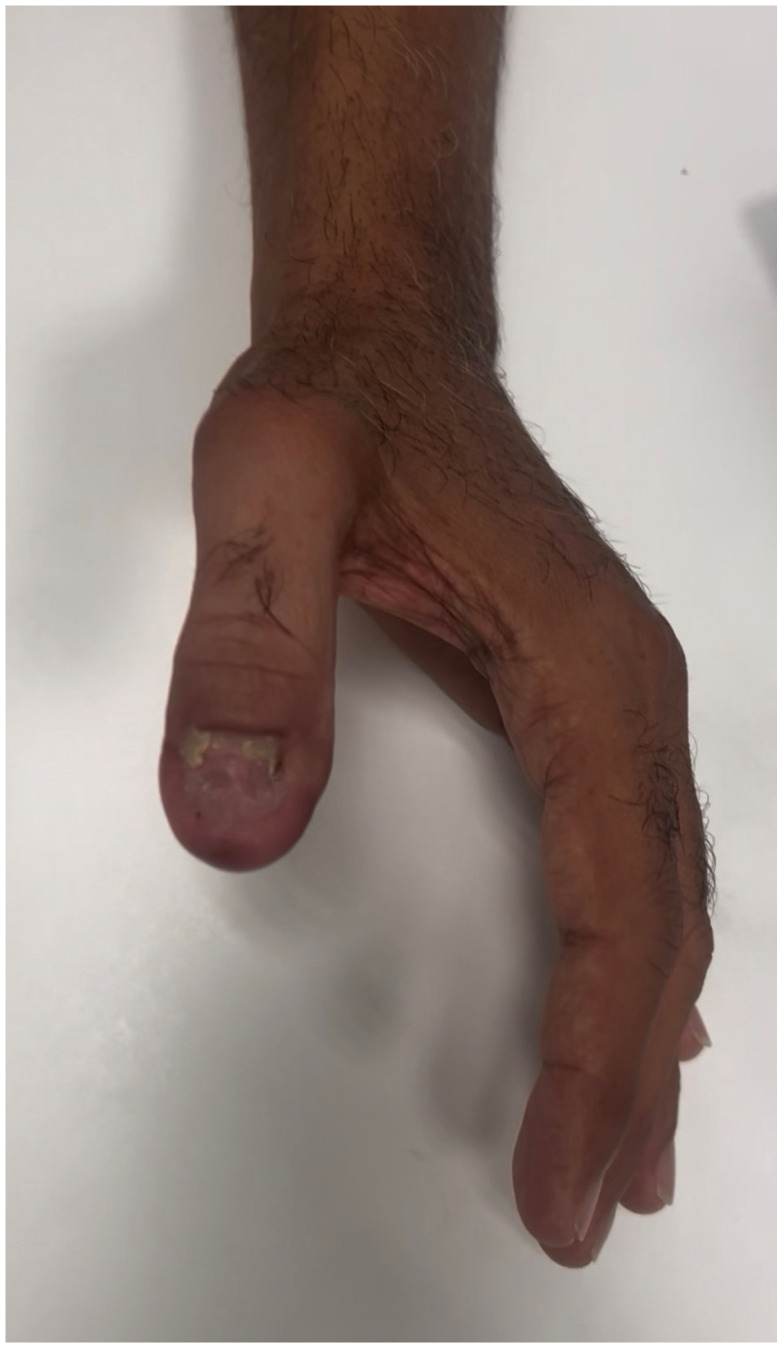
Follow-up after 2 months postoperative, dorsal view.

**Figure 7:**
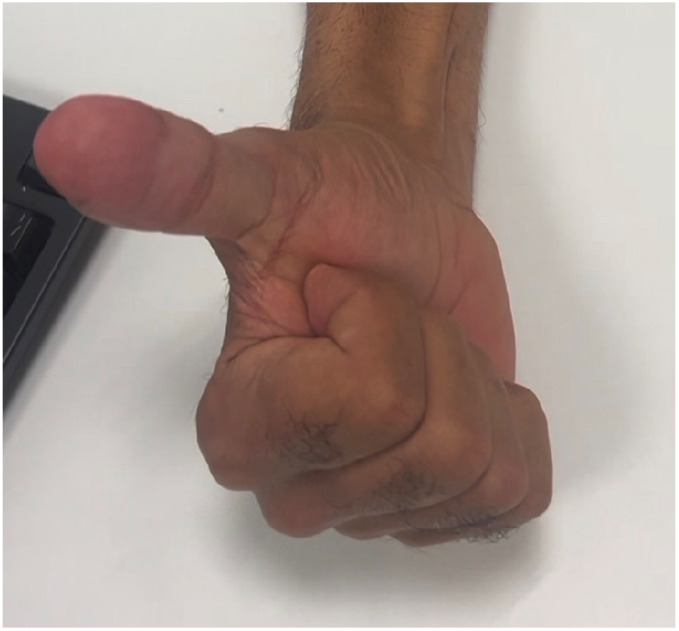
Follow-up after 2 months, volar view.

After six weeks of follow-up, the reconstructed thumb exhibited near-complete integration of the flap, with stable soft-tissue coverage, satisfactory contour, and good tissue pliability. Nail advancement was observed after 3 months, over the adipose-rich dorsal bed, and no secondary procedures were required. Both donor and recipient sites healed without complications (Figure 6 and Figure 7).

### 3.2. Case 2—Using the Fat to Cover the Lateral Incisions

**Figure 8:**
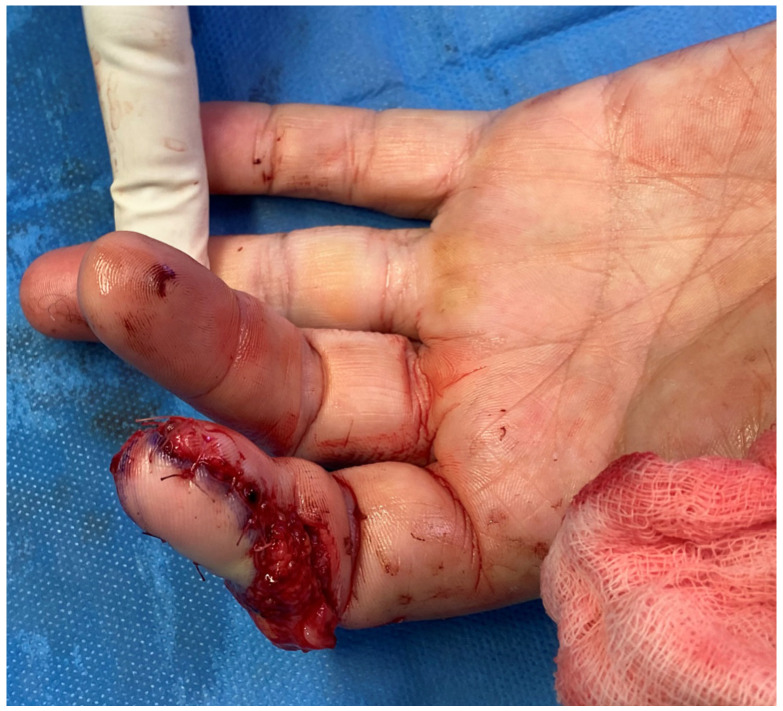
Pulp toe flap for index reconstruction insetting.

**Figure 9:**
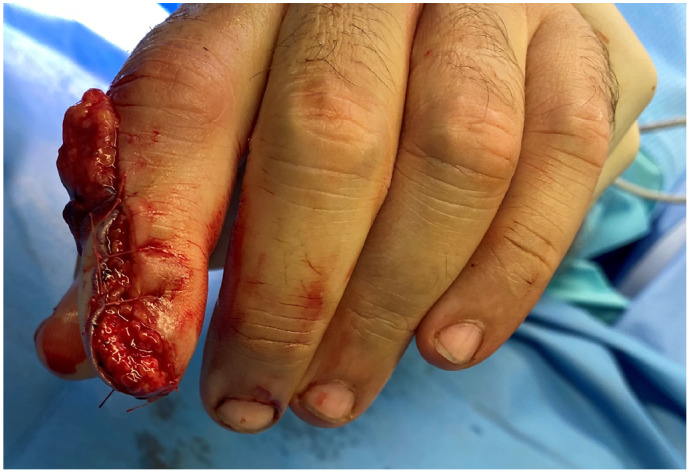
Pulp toe flap for index reconstruction. Fat is used to cover the dorsal part.

**Figure 10:**
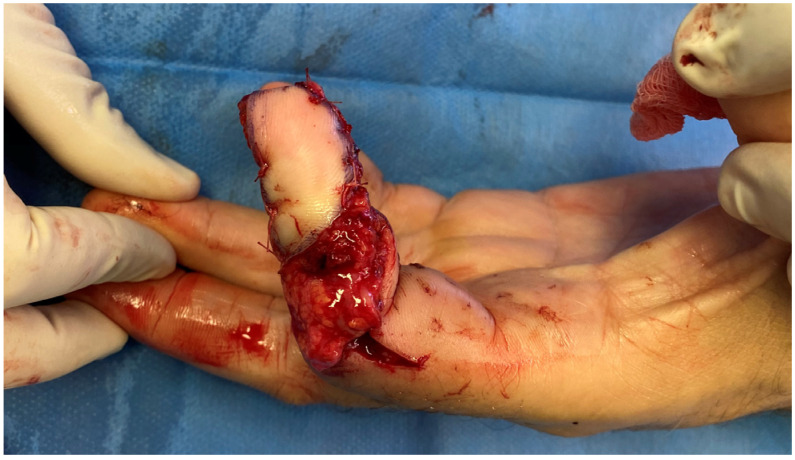
Pulp toe flap for index reconstruction. Fat is used to cover the pedicle without closing the skin.

In all patients, arterial anastomoses were preferentially performed to the digital arteries whenever these vessels were available and demonstrated adequate inflow. Although digital arteries are frequently located close to the zone of injury and may present with small-caliber or intimal damage, distal arterial anastomoses were favored when satisfactory flow was confirmed intraoperatively, in order to avoid unnecessary proximal dissection and additional surgical trauma (Figure 8, Figure 9 and Figure 10).

During flap inset and skin closure, distal skin stiffness and limited tissue compliance occasionally precluded tension-free primary closure. In these situations, excessive closure tension was considered to be potentially hazardous, as it could compromise the vascular pedicle or exert pressure on the distal arterial anastomosis. Rather than forcing primary closure, the wound was intentionally left partially open and covered with a layer of well-vascularized pedicle adipose tissue. This adipose layer served as a protective biological cushion for the anastomoses while maintaining a viable wound bed.

**Figure 11:**
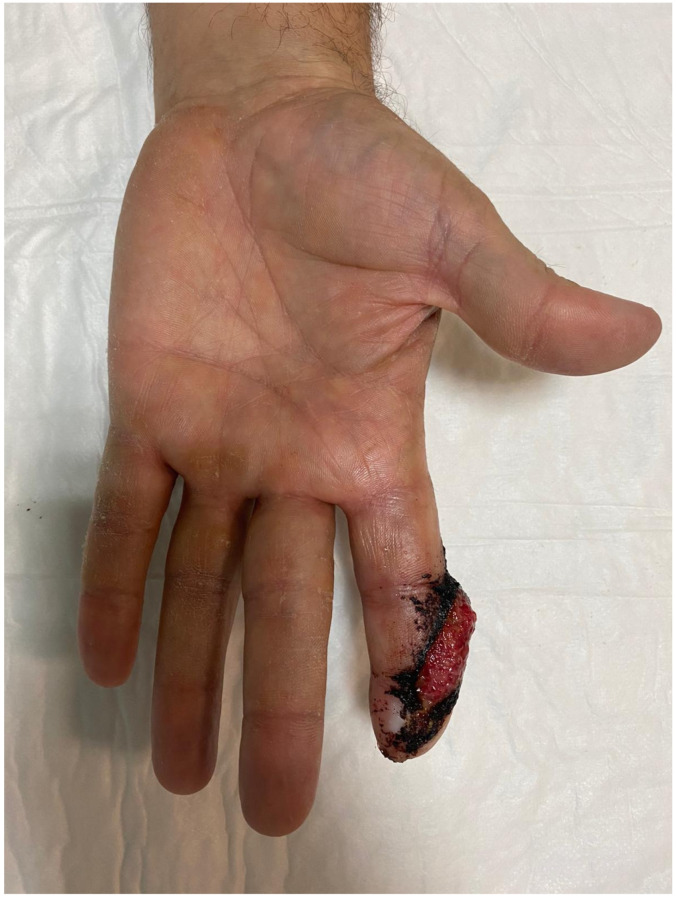
At 3 weeks post-operation, exposed adipose tissue started with granulation and progressive epithelialization.

Postoperatively, the exposed adipose tissue demonstrated predictable granulation and progressive epithelialization (Figure 11).

**Figure 12:**
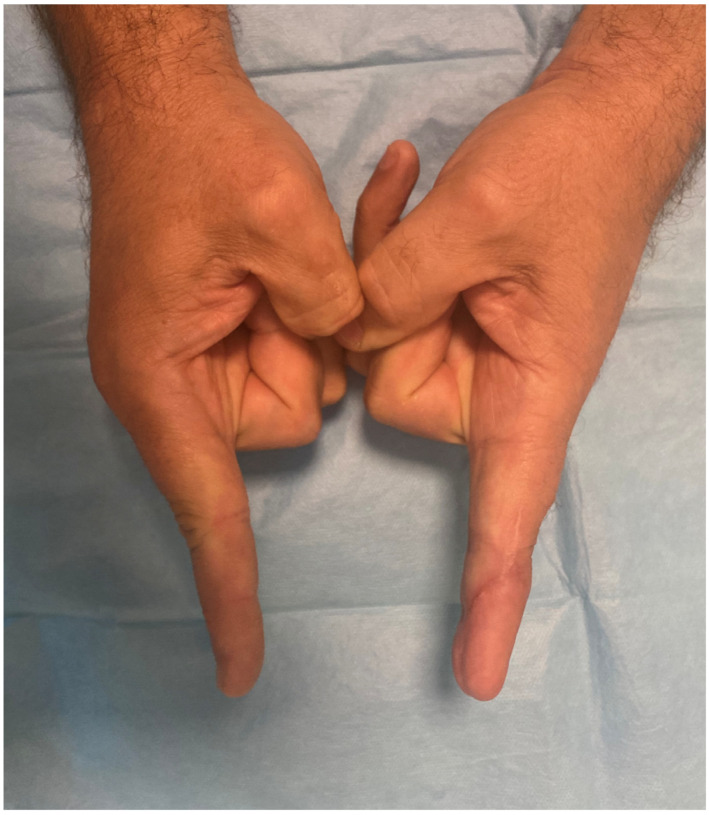
Follow-up after 6 months.

**Figure 13:**
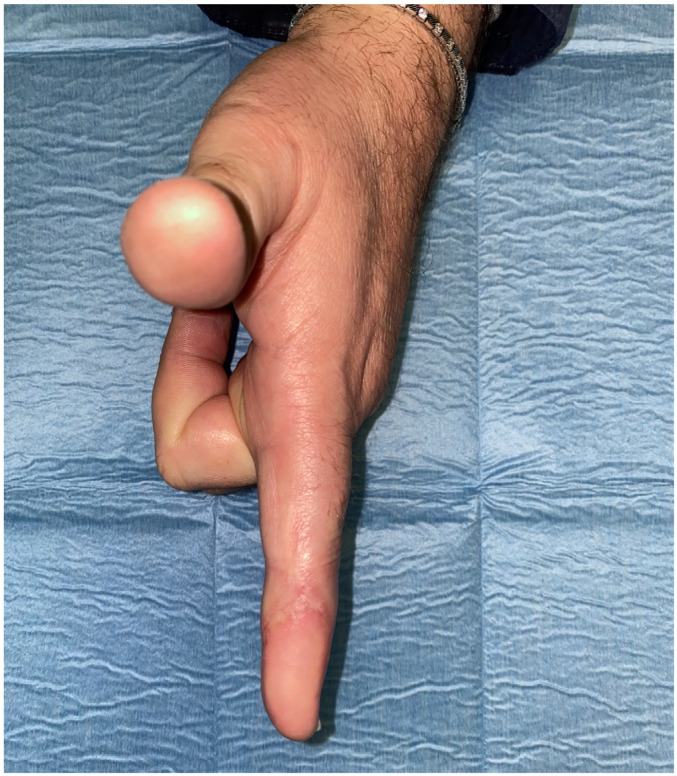
Follow-up after 1 year.

Patients were allowed to initiate hand washing between three and four weeks after surgery without adverse effects on flap survival or wound healing. Complete secondary skin closure was typically achieved within six to seven weeks, resulting in stable soft-tissue coverage with satisfactory cosmetic outcomes. Notably, scars following secondary epithelialization over preserved adipose tissue were often minimal and clinically inconspicuous, and no vascular complications related to delayed closure were observed (Figure 12 and Figure 13).

### 3.3. Case 3—Using the Fat to Reconstruct a Hemi-Trimmed Great Toe

**Figure 14:**
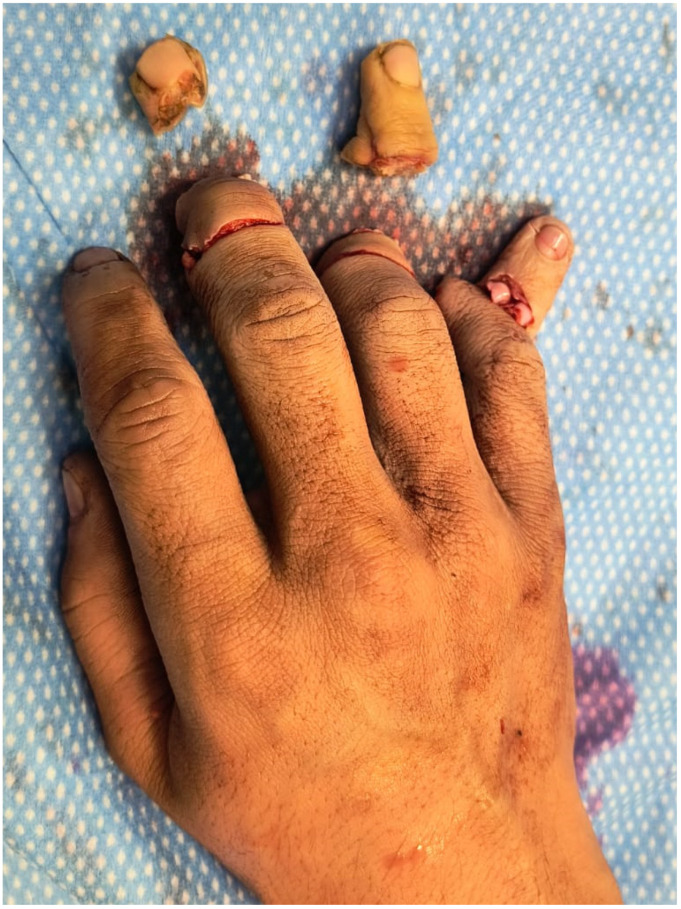
Traumatic amputation of middle, ring and small finger.

An 18-year-old male sustained a severe crush-avulsion injury to the hand involving the middle, ring, and small fingers, following trauma caused by an electric lawn mower. Emergency replantation of all three digits was initially attempted. While replantation of the ring finger was successful, replantation of the distal tip of the middle finger failed, resulting in exposed bone, and the small finger sustained complete loss of the distal phalanx with non-reconstructible soft-tissue damage (Figure 14).

**Figure 15:**
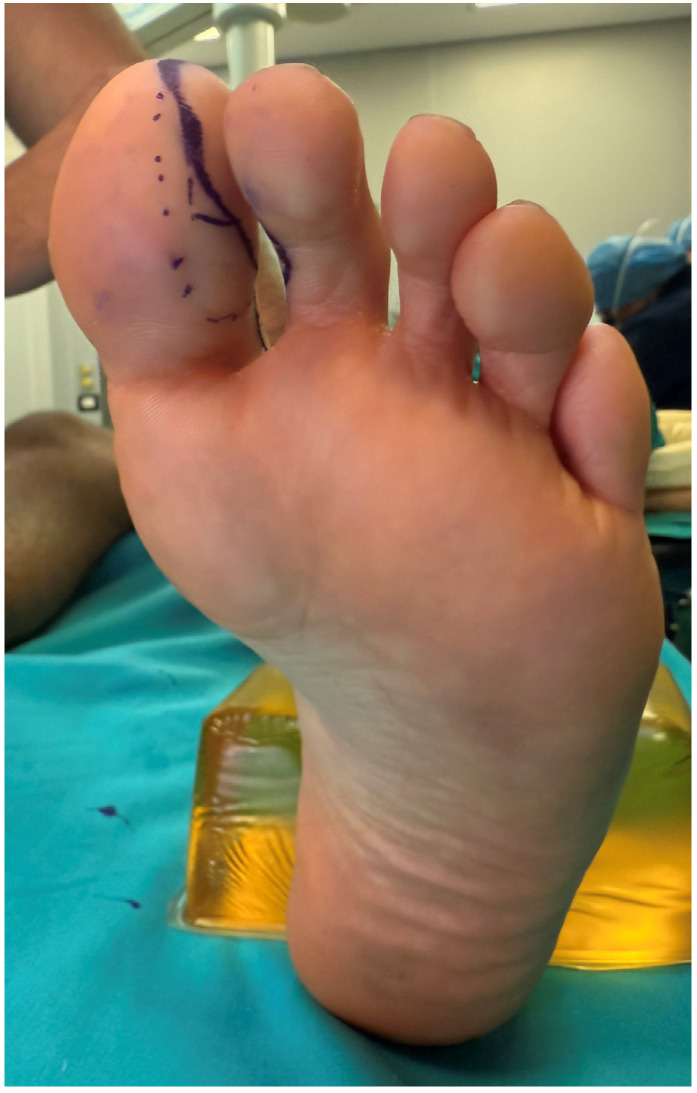
Flap design, including the volar fat pad that will be used to cover the fibular side of the trimmed bone.

**Figure 16:**
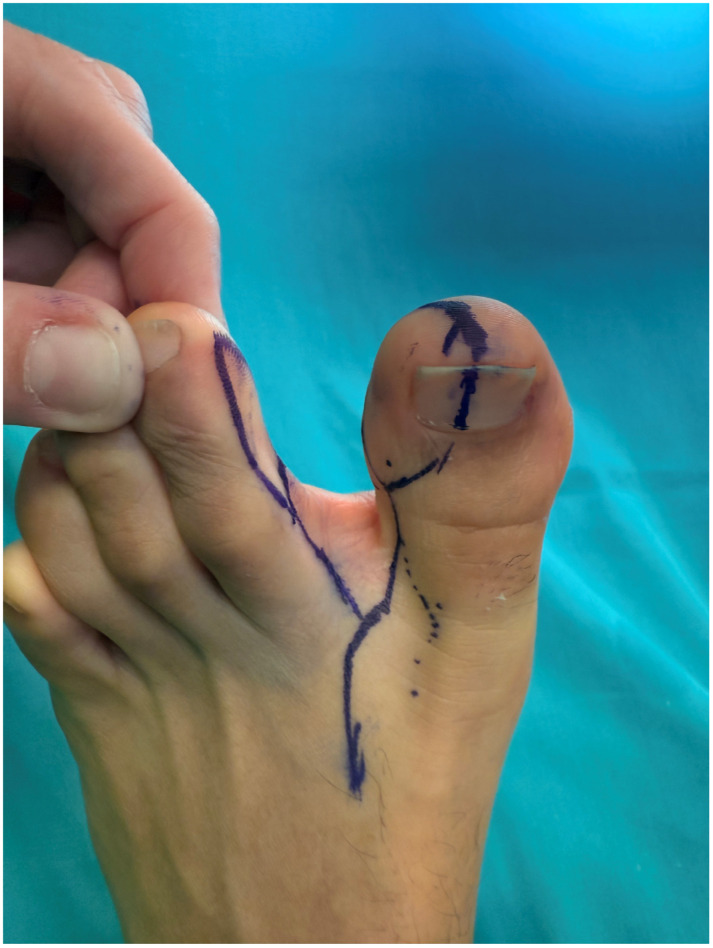
Flap design of the chimeric hemi-trimmed great toe + pulp of the second toe.

Given the extent and heterogeneity of the injuries, a staged reconstructive strategy was planned. Reconstruction of the small finger was performed using a trimmed great toe (TGT) flap, while a chimeric toe pulp flap was used to reconstruct the distal pulp defect of the middle finger. Particular attention was paid to donor-site preservation in order to minimize morbidity and maintain foot esthetics and function (Figure 15 and Figure 16).

**Figure 17:**
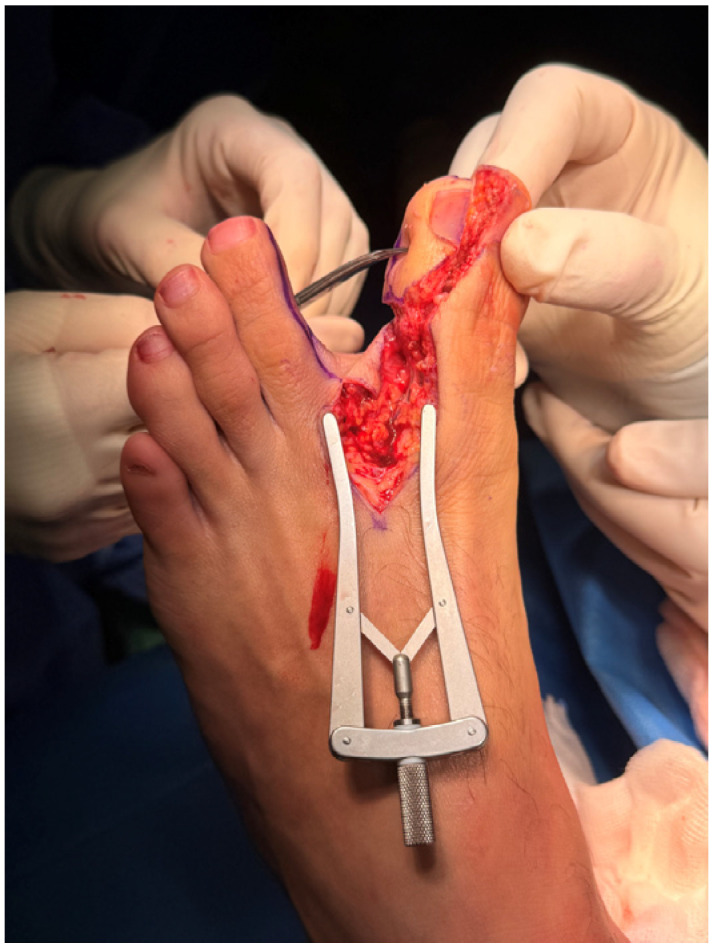
Trimmed great toe.

To avoid complete nail harvest from the donor site and to preserve as much of the great toenail complex as possible, adipose tissue harvested from the fibular aspect of the trimmed great toe was intentionally preserved and mobilized (Figure 17). This adipose component was rotated to reconstruct the volar pad of the small finger and sutured into the neo-nail fold, thereby restoring the soft-tissue volume, contour, and padding while supporting the reconstructed nail complex. Owing to the absence of the distal phalanx and joint destruction, the small finger was stabilized with distal interphalangeal joint arthrodesis to provide a stable and functional digit.

Microvascular anastomoses were performed without difficulty, and flap perfusion was satisfactory. Postoperatively, the adipose tissue demonstrated stable integration without signs of necrosis or infection. The patient resumed ambulation at three weeks without the need for protective footwear. Due to the complexity of the injury and the associated fractures of the middle phalanx of the small finger, rehabilitation of the remaining digits was initiated between three and four weeks post-operation, while mobilization of the small finger began at six weeks.

**Figure 18:**
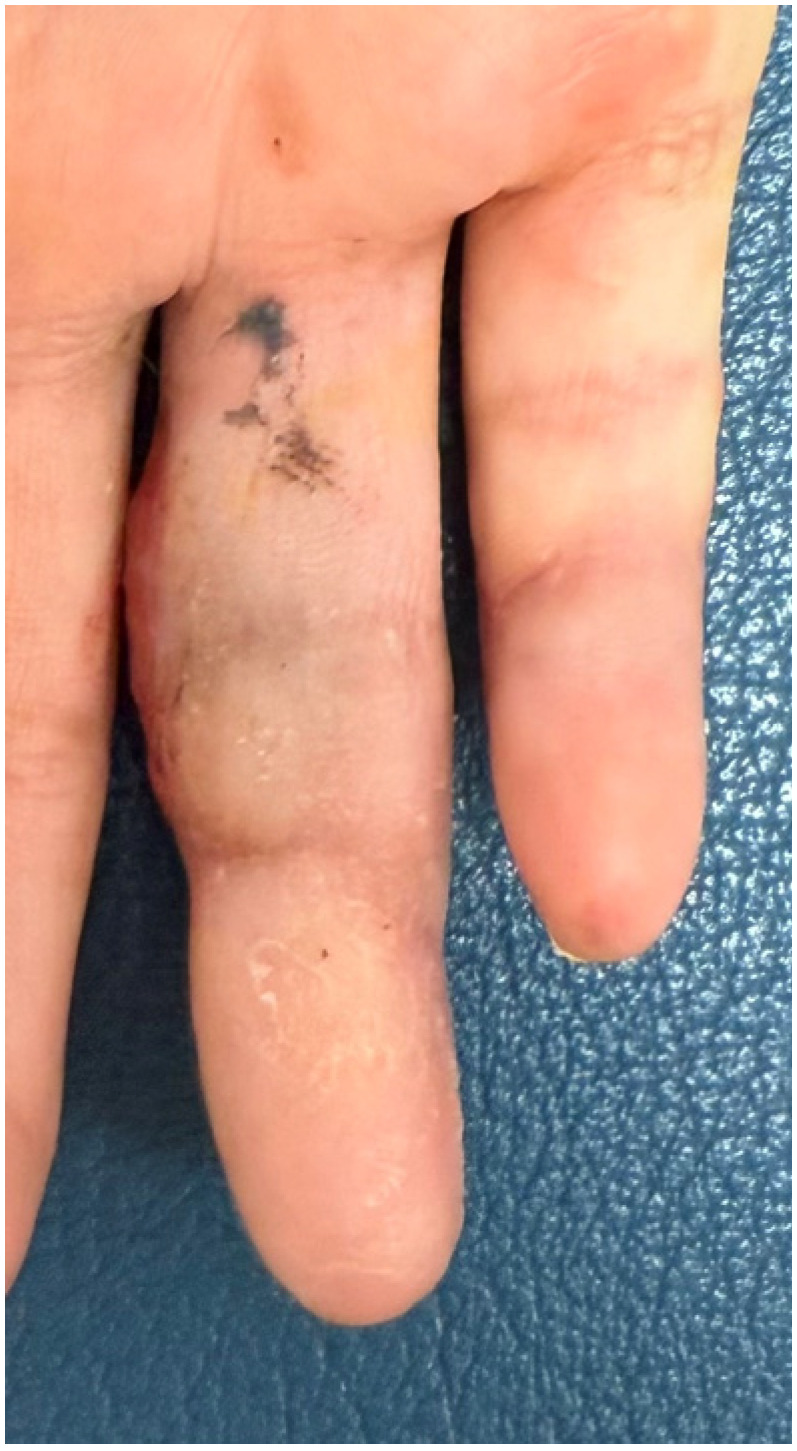
Follow-up of the patient after 10 weeks of closing up the hemi-trimmed great toe.

**Figure 19:**
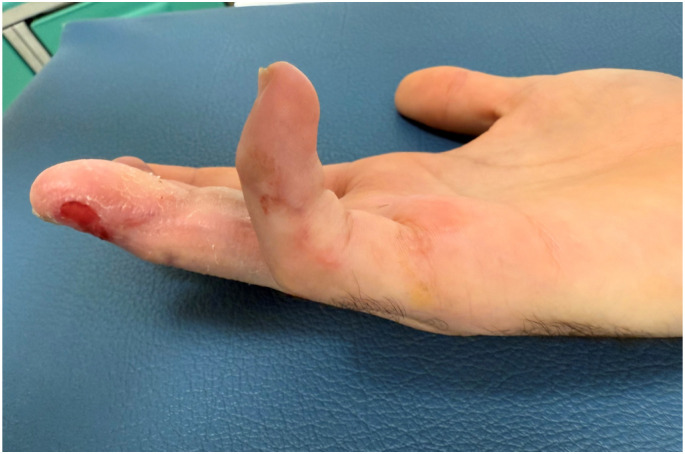
Lateral view of the hemi-trimmed great toe.

At follow-up, both the reconstructed digits and the donor site demonstrated uneventful healing. Adequate padding and contour were achieved at the recipient site, while preservation of a significant portion of the great toenail and volar pad resulted in minimal donor-site morbidity. No complications were observed at either the hand or foot level, illustrating the effectiveness of adipose tissue utilization in complex, multilevel digital reconstruction (Figure 18 and Figure 19).

## 4. Discussion

This study highlights the often underappreciated role of preserved adipose tissue in free flap fingertip reconstruction. While toe-based free flaps are well-established for restoring function, contour, and sensibility in digital defects, the biological behavior of the viable subcutaneous fat, especially when intentionally preserved or left partially uncovered, varies among patients and has received limited attention in the reconstructive literature. Our findings suggest that adipose tissue should not be regarded simply as a passive filler, but rather as an active, patient specific reconstructive component that supports spontaneous healing, contour refinement, and stable soft tissue reconstruction.

Tailoring reconstruction to the individual patient is the goal of every fingertip procedure. The functional and esthetic requirements of a thumb pulp differ substantially from those of the index or a lesser finger, and they are entirely different from those involved in full digit reconstruction. Personalizing the reconstructive strategy according to digit, defect, and patient needs is therefore essential. In this context, the ability to modulate flap design through selective preservation of adipose tissue provides an additional tool to adapt reconstruction to each clinical scenario and to further refine the outcomes [5].

In this large retrospective series, deliberate preservation and strategic use of donor-site adipose tissue were consistently incorporated into the flap design and inset. When primary skin closure was not feasible or would have generated excessive tension over the vascular pedicle, healthy adipose tissue was intentionally left exposed. In these cases, spontaneous epithelialization occurred within a predictable timeframe, with complete healing typically being achieved within 4–6 weeks, without the need for skin grafting. Importantly, this approach did not increase the complication rates at either the recipient or donor site, supporting its safety when the flap perfusion is adequate.

These observations align with classical descriptions of secondary healing in fingertip injuries, in which preserved subcutaneous tissue plays a central role in wound contraction and epithelial migration. However, most of the existing literature on secondary healing focuses on conservative management of small fingertip injuries, rather than microsurgical reconstruction [18,19,20,21]. Our findings extend this principle to free flap reconstruction, suggesting that preserved adipose tissue within vascularized flaps can recreate a biological environment that is conducive to spontaneous healing, even in complex reconstructions.

The biological plausibility of these findings is supported by growing evidence on the regenerative properties of adipose tissue. Adipose-derived stromal cells, together with the intrinsic vascular network of fat, are known to promote angiogenesis, tissue remodeling, and epithelial migration [14,15,16]. In the context of fingertip reconstruction, where soft-tissue quality, pliability, and volume are essential for both function and esthetics, preserved fat appears to act as a biologic scaffold that facilitates secondary healing while maintaining three-dimensional contour [22,23].

Beyond its regenerative role, adipose tissue contributes mechanically to fingertip function. The digital pulp is a specialized fibrofatty structure organized by septa that anchors the skin to the underlying skeleton, allowing for stability during pinch and grasp while dissipating the load [24]. Excessive excision of this tissue may compromise tactile discrimination and comfort. A reconstructive strategy focused on preservation and strategic redistribution of adipose tissue allows for the digit to better approximate native biomechanical and functional behavior.

The three representative cases illustrate the versatility of adipose tissue utilization across different reconstructive challenges. In our experience, adipose tissue was not employed merely as a volumetric filler, but as a dynamic structural and esthetic modulator of distal digital reconstruction. In very distal fingertip injuries, skin coverage alone with the pulp toe flap—particularly when positioned predominantly at the distal margin—may provide patient comfort while failing to adequately guide nail growth, potentially resulting in abnormal curvature of the nail and discomfort for the patient [25,26]. Strategic placement of adipose tissue, both distally and dorsally, creates a compliant, well-vascularized scaffold that supports more physiological nail plate advancement and contour formation. Volar skin coverage, in turn, is prioritized to optimize tactile function and tolerance to shear, reinforcing the functional specialization of the fingertip.

Additionally, intentional preservation and partial exposure of adipose tissue around the vascular pedicle proved to be advantageous in microsurgical settings involving very distal anastomoses. In these scenarios, attempting tight skin closure—particularly following mid-lateral incisions—may increase the risk of pedicle compression and compromise vascular safety. Allowing for controlled secondary epithelialization over viable adipose tissue avoided excessive tension and protected the anastomosis, but also consistently resulted in scars that were subtle and cosmetically acceptable after complete healing after a few weeks.

Finally, selective use of adipose tissue enabled adaptive closure strategies in complex distal phalanx amputations, facilitating contour restoration at the recipient site while simultaneously limiting donor-site morbidity. In toe-pulp-based reconstructions, partial tissue harvest has traditionally been described for the reconstruction of partial digital defects [27,28], whereas complete tissue transfer is typically required for full finger reconstruction [13,29]. In our new approach, partial harvest combined with preservation of adipose and nail components allowed for maintenance of the toe shape and function, avoiding the morbidity associated with more extensive tissue sacrifice.

This tailored use of adipose tissue expands reconstructive flexibility, enhances both esthetic and functional outcomes, and reflects a reconstructive philosophy focused on tissue preservation, rather than maximal harvest. By limiting donor-site tissue removal and leveraging preserved adipose tissue, our approach aligns with contemporary efforts to minimize the functional and esthetic impact on the foot while achieving reliable distal digital reconstruction [30,31].

Importantly, this strategy did not delay postoperative rehabilitation or the initiation of hand washing, both of which are often encouraged to facilitate physiological adaptation to the reconstructed digit and to support sensory re-education. Early sensory input and functional use are known to promote cortical reorganization and to improve long-term sensibility following digital reconstruction. Allowing for early hygiene and controlled use may therefore contribute not only to physical recovery but also to the neural integration of the reconstructed digit [32,33,34].

Several limitations of this study must be acknowledged. Its retrospective design and lack of a control group preclude a direct comparison with alternative reconstructive strategies. Functional outcomes were assessed clinically, rather than through validated patient-reported outcome measures, limiting quantitative evaluation of patient satisfaction and sensory quality. Additionally, while the healing patterns were consistent, histological or molecular analysis of the healing process was beyond the scope of this study.

Despite these limitations, the strength of this study lies in the large number of patients, the consistency of the surgical technique, and the reproducibility of healing outcomes across different flap types. The uniform observation of spontaneous epithelialization over preserved adipose tissue supports the concept that viable fat plays an active role in reconstruction, rather than representing expendable tissue.

## 5. Conclusions

In summary, this retrospective case series suggests a conceptual shift in free flap fingertip reconstruction, in which preserved adipose tissue may be regarded as an active biological asset, rather than tissue to be discarded or fully buried. Strategic utilization of donor-site fat may simplify reconstruction and potentially reduce the need for secondary procedures. Because adipose tissue exhibits patient-specific biological and regenerative behavior, its preservation supports a more personalized approach to fingertip reconstruction. By recognizing adipose tissue as a regenerative adjunct—the “forgotten healer”—our findings support the hypothesis that tissue-preserving, individualized strategies can refine reconstructive decision-making and improve outcomes in distal digital reconstruction.

## Data Availability

The data supporting the findings of this study are not publicly available due to privacy and ethical restrictions related to patient confidentiality, but they can be made available from the corresponding author upon reasonable request.
